# Comparison Between the Advanced Cardiac Life Support and Adult Advanced Life Support Protocols: A Simulation-Based Pilot Study

**DOI:** 10.1155/2024/6696879

**Published:** 2024-10-18

**Authors:** Fawaz Altuwaijri, Abdulaziz Alrabiah, Abdullah Alqarni, Alia Kamal Habash, Mohammad Alghofili, Omar Alotaibi, Mansour Altuwaijri

**Affiliations:** ^1^Department of Emergency Medicine, College of Medicine, King Saud University, Riyadh, Saudi Arabia; ^2^Division of Gastroenterology, Department of Medicine, College of Medicine, King Saud University, Riyadh, Saudi Arabia

**Keywords:** American, Australian, cardiopulmonary, heart association, resuscitation, resuscitation council

## Abstract

**Introduction:** Cardiac arrest is a public health concern associated with unfavorable disease outcomes. Cardiopulmonary resuscitation (CPR) of optimal quality is widely acknowledged as an indispensable technique in restoring spontaneous circulation. In order to perform advanced cardiac life support (ACLS), chest compression must be paused twice: once to assess the rhythm and again to administer the shock. Australian advanced life support (ALS) recommends that the defibrillator needs to be precharged in order to administer the shock during a solitary interval in chest compressions. While performing chest compressions, precharging defibrillators can decrease hands-off time without posing a risk of injury.

**Aim:** To compare chest compression fraction (CCF)—which is the cumulative time spent providing chest compressions divided by the total time taken for the entire resuscitation—by calculating the hands-off time duration in cardiac arrest between the Australian Resuscitation Council (ARC) and American Heart Association (AHA) protocols for CPR.

**Methods:** A simulation-based pilot study was designed using a Laerdal Resusci Anne mannequin and a LIFEPACK 20 defibrillator. The study included six participants recruited from King Khalid University Hospital in Riyadh, Saudi Arabia, where three participants were certified ACLS providers and there were certified ALS providers. Participants were divided into two groups, ALS and ACLS, each following one protocol. For each scenario, a random job was assigned to each participant, regardless of their role as assistant, team leader, or performer of CPR. Each case's shockable and nonshockable rhythms were hidden from the team leader and the chest compressor. Ten trials of CPR were performed, each for four cycles with a total time of 8 min. The simulation was video recorded for hands-off time counting. Comparison between CCF (seconds) per cycle between the two protocols was performed using an independent sample *t*-test. A *p* value of 0.05 was used to measure statistical significance.

**Results:** Comparing CCF in shockable rhythms between ARC and AHA protocols, it was observed that the CCF of ALS-ARC was significantly higher than ACLS-AHA in all cycles; the first cycle: *t* = 3.782, *p*=0.004; the second cycle: *t* = 3.380, *p*=0.007; the third cycle: *t* = 3.803, *p*=0.003; and the fourth cycle: *t* = 4.341, *p*=0.001.

**Conclusion:** Precharging a defibrillator before a rhythm check during chest compression, in anticipation of a potentially shockable rhythm, reduces the time required for defibrillation and limits interruptions in chest compression during CPR. This practice effectively enhances the CCF. Enhancing the continuity of chest compressions can potentially improve survival rates in ARC.

## 1. Introduction

Cardiac arrest is a significant public health issue, with a majority of patients experiencing unfavorable prognoses [1]. The definition of high-quality cardiopulmonary resuscitation (CPR) encompasses several key components. These include a chest compression fraction (CCF) exceeding 80%, a compression rate ranging from 100 to 120 compressions per minute, a compression depth of at least 50 mm (equivalent to 2 inches), and no excessive ventilation [2]. The delivery of blood to major organs at an appropriate level of coronary perfusion pressure, as achieved through high-quality CPR, is widely acknowledged as a crucial mechanism for achieving the return of spontaneous circulation (ROSC) and a positive outcome in cases of cardiac arrest that occur outside of a hospital setting [3]. More recent guidelines from the American Heart Association (AHA) and Australian Resuscitation Council (ARC) for CPR prioritize enhancing the quality of CPR in order to enhance survival rates [4].

Improving survival rates for cardiac arrest patients depends on multiple chest compression characteristics such as correct chest compression depth and rate, adequate chest recoil, and, most importantly, minimizing interruptions. A CCF—the ratio of the cumulative duration allocated to administering chest compressions to the overall duration, including the complete resuscitation process—of 60% and above has been shown to increase the survival rate significantly. Interruptions are usually the time taken to check rhythm, pulses, intravenous cannulation, intubation, and delivering a shock, if needed, to a person with shockable cardiac arrest [2]. High-quality chest compressions achieve a cardiac output of about 30% of the average value. Chest compression interruptions have been shown to decrease cardiac output and cerebral and coronary perfusion pressures [5], which leads to poor prognosis and higher mortality from cardiac arrest.

A study reports that high-quality CPR, early defibrillation, and minimizing hands-off time (i.e., the total number of pauses in chest compression for each cycle of CPR) can enhance survival rates [6]. It has previously been observed that a shorter hands-off time increases the odds of survival [7]. The AHA highlighted the need to reduce interruptions during chest compression cycles to improve the quality of chest compressions and attain more favorable outcomes [2]. Few studies evaluated the role of perishock pause (defined as the time consumed before- and after-the shock delivery); however, their results are inconsistent. Some animal studies showed that shorter perishock interruptions during chest compression improved the outcomes [8].

**Figure 1:**
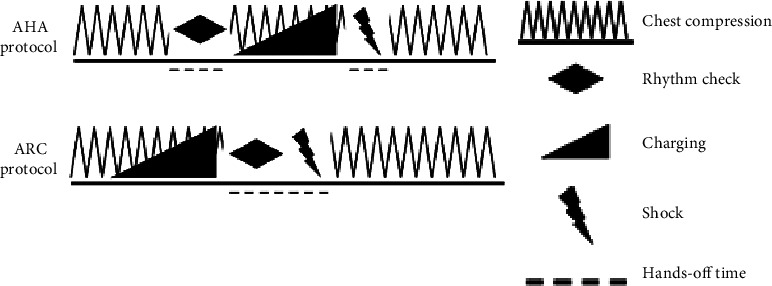
Illustration of the differences between AHA and ARC protocols.

The advanced cardiac life support (ACLS) by AHA guidelines states that the rescuer should initiate CPR, pause to analyze rhythm, and resume CPR while the defibrillator is charging. Once the defibrillator is charged and ready to deliver the electrical shock, the rescuer should pause chest compressions for the second time to deliver the shock and continue CPR afterward [9]. On the other hand, the adult advanced life support (ALS) guidelines provided by the ARC advocate for the prompt charging of the defibrillator during chest compressions. This ensures that the defibrillator is prepared and fully charged if a rhythm check is required. The main difference between the two protocols is dealing with shockable rhythms, where the ACLS guidelines stops chest compressions twice before delivering the shock and the ALS stops chest compressions once before the shock is delivered. On the other hand, both protocols stop CPR once when the rhythm is analyzed as “nonshockable rhythm.” Both protocols are shown in Figure 1.

Numerous research studies, including both human subjects and mannequins, have been conducted to assess the effectiveness of charging the manual defibrillator while performing chest compressions, prior to interrupting the compressions to assess the cardiac rhythm. Whether a shockable heart rhythm is detected, the shock either commences to the patient or the defibrillator is disarmed. They found that the precharging method reduces the number of pauses and hands-off time [10–12]. However, no study has assessed the impact of the precharging approach on clinical outcomes in cardiac arrest cases or improvement in survival rates [13].

Previous research has established that precharging the defibrillator reduces hands-off time without risking harm during chest compression [10]. However, this is the first study comparing hands-off time duration in cardiac arrest between the ARC and AHA protocols.

## 2. Methodology

A comparison study was conducted in a medical simulation center utilizing simulation-based methods. The trials used a Laerdal Resusci Anne mannequin and a LIFEPACK 20 defibrillator (Physio-Control Inc., WA, USA). Six participants were recruited from King Khalid University Hospital (KKUH) in Riyadh, Saudi Arabia. Ten simulated patient scenarios with cardiac arrest were performed, each for four cycles with a total time of 8 min. The simulation was video recorded for hands-off time counting. Participants' allocation was based on ALS licenses, either ARC or AHA. The participants were categorized into two groups, consisting of three individuals each. The first group adhered to the protocol established by the ARC, while the second group followed the guidelines set by the AHA. Every participant was assigned to a specific assignment for each scenario randomly, including roles such as team leader, CPR performer, or defibrillator assistant.

**Figure 2:**
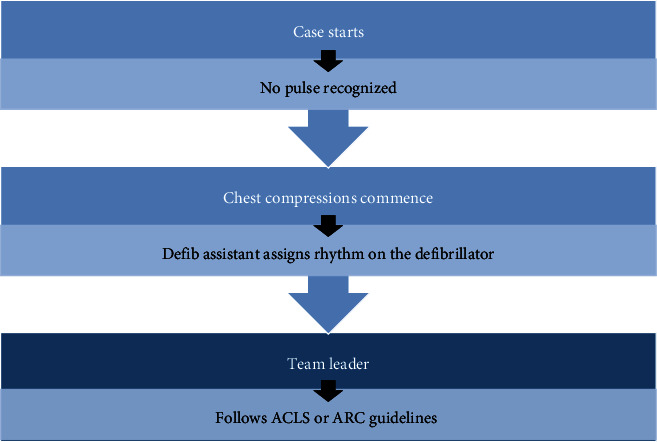
Schema of participants' flow.

The team leader and CPR performer were blinded to the cardiac rhythm to control for bias. Each case started with a short patient history to simulate real cases. The team leader was responsible for command and rhythm checks. The defibrillator assistant handled ventilation, defibrillation, and drug administration. The defibrillator assistant kept 10 cards with different rhythms (asystole, pulseless ventricular tachycardia, ventricular fibrillation (VF), and pulseless electrical activity) and randomly assigned the rhythm according to the card picked.  Figure 2 shows the schema of participants' flow and characteristics.

### 2.1. Statistical Analysis

The mean and standard deviation were utilized in the presentation of descriptive statistics. A comparison was made between the ARC and AHA protocols utilizing an independent sample *t*-test to examine the CCF (seconds) in each cycle. The level of statistical significance was determined by using a *p* value of 0.05. Statistical Packages for Social Sciences Version 26 (SPSS, Armonk, New York, IBM Corporation) was used for every one of the studies performed on the data.

## 3. Results

Comparison of CCF between AHA and ARC protocols is tabulated in Table 1, where statistical significance was reached between the protocols (*p*=0.0026) and their shockable rhythms (*p*=0.0005).  Table 2 compares the time off-chest (seconds) between ARC and AHA protocols. Based on the results, the mean time duration of time off-chest (seconds) in ACLS-AHA in all cycles was longer than ALS-ARC. However, statistical tests yielded that the comparison of time off-chest (seconds) did not reach statistical significance (all *p* > 0.05).

Table 3 compares the time off-chest in shockable rhythms between ARC and AHA protocols. It can be observed that the time off-chest (seconds) of ACLS-AHA was significantly longer than ALS-ARC in all cycles; the first cycle: *t* = 3.782, *p*=0.004; second cycle: *t* = 3.380, *p*=0.007; third cycle: *t* = 3.803, *p*=0.003; and fourth cycle (*t* = 4.341; *p*=0.001). Comparing the time off-chest in nonshockable rhythms between ARC and AHA protocols, it was found that the time off-chest (seconds) in all cycle levels was not significantly different in both groups (*p* > 0.05). This nonsignificant difference in results in the nonshockable rhythms is believed to be the outcome of the fact that both protocols treat nonshockable rhythms similarly, where chest compressions are paused once before “dumping the charge” and resuming chest compressions (Table 4).

## 4. Discussion

To the best of our current understanding, this study represents the first simulation-based pilot investigation that compares the duration of hands-off periods between the ACLS-AHA and ALS-ARC protocols. The key finding of the study is that the CCF is considerably influenced by the defibrillation approach. We have found that the ARC protocol has a significantly shorter time on all cycles of shockable rhythm, with a mean of fewer than 6.7 s and a *p* value of ≤ 0.05. In accordance with the present results, Edelson et al. [10] demonstrated a notable reduction in total hands-off time by using the charging before rhythm analysis method in comparison with the AHA method in the presence of shockable rhythm (3.9 s (2.4–5.6) vs. 11.5 s (9.1–14.5); *p*  <  0.001).

The results of this study provide more support for the justification of endeavors aimed at minimizing the duration of defibrillation in order to optimize the efficacy of resuscitation in individuals experiencing VF or pulseless ventricular tachycardia.

A summary of the statistics for both shockable and nonshockable rhythms mean of CCF is shown in Table 1. Although not statistically significant, the mean duration of ACLS-AHA was longer in all cycles. A similar pattern of results was obtained from previous studies [10–12]. In the nonshockable group, no major differences were found between the two protocols regarding duration, and none were statistically significant. Both protocols do not affect hands-off time; a possible explanation might be that nonshockable rhythms have only one pause.

According to these data, we can infer that anticipation of a potentially shockable rhythm can reduce the total hands-off time. Although the incidence of VF appears to be decreasing, it remains a common initial rhythm encountered in cardiac arrest patients [14], owing largely to improved treatment and prevention of coronary artery disease and its complications [15]. Early defibrillation is the most effective method for restoring spontaneous circulation after cardiac arrest caused by VF [14].

Previous research established that prolonged hands-off time results in a considerable rise in mortality rate [16, 17]. In practice, CPR is typically carried out with prolonged pauses in chest compression, regardless of resuscitation guideline recommendations for minimizing chest interruption [16].

Charging during ongoing chest compression may raise concerns about the technique's safety. In a previous study, over 354 shocks were commenced, with only one observed incident where the shock was commenced while rescuers performed chest compression. Compression remained at the same rate, which suggested that no impact had been inflicted on the rescuer [10].

According to our knowledge, the present study is a first-of-its-kind comparative analysis conducted as a pilot study to evaluate the efficiency of two widely accepted cardiac life-support protocols. The comparison of ALS and ACLS clearly showed that ALS-ARC protocols might have better outcomes. These findings correlate with previous studies and are supported by published data. However, more comparative research will be required to determine the influence of these protocols on patient outcomes.

Our study carries certain limitations, including its single-center setting and simulation-based design hampering the measurement of mortality and morbidity. Another limitation is the lack of means to directly measure coronary perfusion pressure while performing CPR.

## 5. Conclusion

Precharging defibrillation during chest compression, in anticipation of a shockable rhythm at the rhythm check, reduces the time required for defibrillation and minimizes interruptions in chest compression during CPR. The reduction of interruptions during chest compression is a critical factor in enhancing outcomes in CPR, with particular emphasis placed on minimizing the duration of the longest interruptions. The findings of our study indicate a significant correlation between delays and a decline in CCF, whereby each extra minute of delay is linked to a decrease in survival outcomes. Nevertheless, it is imperative to replicate the findings of this study in a more extensive cohort of patients or, alternatively, conduct further investigations to validate the results before proceeding with replication in a larger cohort.

## Figures and Tables

**Table 1:** Comparison of CCF between AHA and ARC protocols.

	CCF in %		*t*-test	*p* valuea
	ACLS-AHA	ALS-ARC		
	Mean ± SD	Mean ± SD		
All cases	93.08 + 0.76	94.58 + 0.37	4.1206	**0.0026** b
Shockable rhythms	91.39 + 0.49	93.82 + 0.29	8.1557	**0.0005^b^**
Non-shockable rhythms	95.62 + 0.38	95.73 + 0.22	0.5034	0.6493

*Note:* Bold = significant *p* value.

Abbreviations: ACLS = advanced cardiac life support; AHA = American Heart Association; ALS = advanced life support; ARC = Australian Resuscitation Council; SD = standard deviation.

^a^ *p* value has been calculated using an independent sample *t*-test.

^b^Significant at *p* ≤ 0.05 level.

**Table 2:** Comparison of time off-chest in all rhythms between AHA and ARC protocols.

Cycle level	Time off-chest in seconds		*t*-test	*p* valuea
	ACLS-AHA	ALS-ARC		
	Mean ± SD	Mean ± SD		
First cycle	7.80 ± 2.97	6.20 ± 1.39	1.540	0.141
Second cycle	9.00 ± 3.23	6.70 ± 1.64	2.008	0.060
Third cycle	8.60 ± 2.99	6.70 ± 1.49	1.798	0.089
Fourth cycle	7.80 ± 2.78	6.40 ± 1.35	1.432	0.169

Abbreviations: ACLS = advanced cardiac life support; AHA = American Heart Association; ALS = advanced life support; ARC = Australian Resuscitation Council; SD = standard deviation.

^a^ *p* value has been calculated using an independent sample *t*-test.

**Table 3:** Comparison of time off-chest in shockable rhythms between AHA and ARC protocols.

Cycle level	Time off-chest in seconds		*t*-test	*p* valuea
	ACLS-AHA	ALS-ARC		
	Mean ± SD	Mean ± SD		
First cycle	9.83 ± 1.33	7.00 ± 1.26	3.782	**0.004^b^**
Second cycle	11.2 ± 2.14	7.67 ± 1.37	3.380	**0.007^b^**
Third cycle	10.7 ± 1.75	7.67 ± 0.82	3.803	**0.003^b^**
Fourth cycle	9.67 ± 1.21	7.33 ± 0.52	4.341	**0.001^b^**

*Note:* Bold = significant *p* value.

Abbreviations: ACLS = advanced cardiac life support; AHA = American Heart Association; ALS = advanced life support; ARC = Australian Resuscitation Council; SD = standard deviation.

^a^ *p* value has been calculated using an independent sample *t*-test.

^b^Significant at *p* ≤ 0.05 level.

**Table 4:** Comparison of time off the chest in nonshockable rhythms between AHA and ARC protocols.

Cycle level	Time off-chest in seconds		*t*-test	*p* valuea
	ACLS-AHA	ALS-ARC		
	Mean ± SD	Mean ± SD		
First cycle	4.75 ± 1.71	5.00 ± 0.00	−0.293	0.780
Second cycle	5.75 ± 0.50	5.25 ± 0.50	1.414	0.207
Third cycle	5.50 ± 0.58	5.25 ± 0.96	0.447	0.670
Fourth cycle	5.00 ± 1.83	5.00 ± 0.82	0.000	1.000

Abbreviations: ACLS = advanced cardiac life support; AHA = American Heart Association; ALS = advanced life support; ARC = Australian Resuscitation Council. SD = standard deviation.

^a^ *p* value has been calculated using an independent sample *t*-test.

## Data Availability

The datasets generated during and/or analyzed during the current study are available from the corresponding author upon reasonable request.
